# Warren on the Excision of the Tonsils

**Published:** 1842-10-15

**Authors:** 


*Excision of the Tonsils.*—Dr. J. Mason Warren read the following paper to the Boston Society for Medical Improvement, Aug. 22d, 1842.

In 1839 I presented to this Society some remarks on enlargement of the tonsils, attended by a certain deformity of the chest, and on the operation which had been practised for their relief.* Since that time I have had occasion to perform this operation on between one and two hundred patients. The results have led me to the same conclusion as stated at that time. I regret that I heve not kept a sufficiently accurate account of all these cases to

* Published in the Medical Examiner, Vol. II., No. 20, p. 309.

be able to lay them before the Society. Within the last two months I have had occasion to perform this operation of excision of the tonsils on 13 patients. Eleven of these were children. In seven of the children the sternum was excavated or otherwise deformed. Six of the 13 patients were deaf. One immediately recovered his hearing after the operation; in the others some days elapsed before the improvement took place. In all of them the breathing was more or less affected, and relief almost immediately followed the operation.

In a late number of the Medical Examiner, I observed some remarks made by a distinguished surgeon in Philadelphia, in regard to a certain change of voice following on this operation, and suggested as the probable cause the injury done to the lateral half arches of the palate by the operation, from adhesions existing between them and the enlarged tonsils. I have taken some pains, since those remarks came under my observation, to examine a number of patients on whom I had previously operated, and in none of them could I observe, or had they observed themselves, the change of voice alluded to. In none of the 13 cases now given did this alteration exist.

There are few of the operations in surgery in which the relief is so immediate and so permanent, as in this simple one of excision of the tonsils, and the results, so far as I have observed, are uniformly favourable.

The instrument which I have most commonly used has been the guillotine instrument, with a convex instead of a spear-pointed blade. The broad shoulder of the instrument, which has been sometimes objected to, serves in the place of a spatula to hold the tongue well down, and the edges of the ring which embrace the tonsil, if not too to narrow, press back the columns of the palate, and protect them from being wounded.—*New Eng. Journ. of Med. and Surg. Oct.*, 1842.

